# Reducing Pharmaceuticals in Water, a New Module Integrated in the Pharmacy Game: Evaluating the Module’s Effects on Students’ Knowledge and Attitudes

**DOI:** 10.3390/pharmacy12010028

**Published:** 2024-02-06

**Authors:** Tanja Fens, Caroline T. A. Moermond, Peter van der Maas, Claudia Dantuma-Wering, Geke H. Lestestuiver, Agata Szperl, Lisette C. M. Schuiling, Eelko Hak, Katja Taxis

**Affiliations:** 1 Unit of PharmacoTherapy, -Epidemiology and -Economics, Groningen Research Institute of Pharmacy and School of Science and Engineering, University of Groningen, Antonius Deusinglaan 1, 9713 AV Groningen, The Netherlandse.hak@rug.nl (E.H.); k.taxis@rug.nl (K.T.); 2 Center for Safety of Substances and Products, National Institute for Public Health and the Environment (RIVM), P.O. Box 1, 3720 BA Bilthoven, The Netherlands; caroline.moermond@rivm.nl; 3 Research Group Sustainable Water Systems, Van Hall Larenstein, University of Applied Sciences, Agora 1, 8901 BV Leeuwarden, The Netherlands; 4 Unit of Safety and Environment, University Medical Center Groningen, Hanzeplein 1, 9713 GZ Groningen, The Netherlands; 5 Centre for Learning and Teaching (CLT), School of Science and Engineering, University of Groningen, Antonius Deusinglaan 1, 9713 AV Groningen, The Netherlands; a.m.szperl@rug.nl; 6 Behavioural and Social Sciences, University of Groningen, Grote Kruisstraat 2/1, 9712 TS Groningen, The Netherlands

**Keywords:** pharmaceuticals in water, eco-toxicity, environmental sustainability, pharmacy education, the pharmacy game, e-learning

## Abstract

Pharmaceutical residues end up in surface waters, impacting drinking water sources and contaminating the aquatic ecosystem. Pharmacists can play a role in reducing pharmaceutical residues, yet this is often not addressed in pharmacy undergraduate education. Therefore, we developed the educational module “Reducing Pharmaceuticals in Water” for pharmacy students; this was integrated in our pharmacy simulation game for third year Master of Pharmacy students at the University of Groningen. In this study, we aim to evaluate the effects of the module on students’ knowledge of pharmaceutical residues in water, to describe students’ experiences in taking the module, and to explore their attitudes towards green pharmacy education in general. This mixed-methods study included quantitative measurements, before and after students took the module (intervention group) and in a control group which did not receive the module. Data were collected between February 2023 and June 2023. Overall, 29 students took the module and 36 students were in the control group. The knowledge score of students in the intervention group (*N* = 29) increased significantly from 9.3 to 12.9 out of 22 (*p* < 0.001). The knowledge score of the students in the control group was (8.9 out of 22). Students found the e-learning and the patient cases the most exciting part of this module. Students also recognized the need to including environmental issues in pharmacy education. In conclusion, the module contributes towards improved knowledge and increased awareness of the impact of pharmaceuticals found in water. It represents a promising strategy to strengthen pharmacist’s role in mitigating the amount and the effect of pharmaceuticals on water and the environment in the future.

## 1. Introduction

There is a global trend of increasing production and consumption of pharmaceuticals, which is tightly related to new research and technology developments, but also to the aging of the population. Increased concentrations of pharmaceuticals or their transformation products are found in various environmental matrices such as water (waste-, surface, ground-, drinking water, or sediments), the soil (agricultural soil or land fields), or the air, raising concerns about their potential impact on eco-systems, aquatic life and human health [1,2,3]. A study undertaken in five UN regions (71 countries around the world) showed the presence of 16 pharmaceuticals or their transformation products indicating urban wastewater as the dominant emission pathway [4]. Moreover, pharmaceutical production and usage sites (industry and hospitals), wastage from unused pharmaceuticals by patients and excretion by patients are seen as the main sources of pharmaceutical residues. The current wastewater treatment plants are not designed to remove pharmaceutical residues with removal percentages around 60%, ranging from 0 to 100% depending on the specific substance [1]. Various factors influence the effectiveness of removing the pharmaceuticals from the wastewater. These include, for example, the substance properties (how soluble, absorbent or stable the substance is), the nature of the substance (organic or non-organic origin, temperature, pH values), substance concentration, wastewater treatment plant characteristics (what kind and how advanced the removal technology is) and finally how these all are regulated by the local or national governance in one geographical area. As this may vary from one place to another, a combination of techniques and approaches may be required to address this complex issue properly. Current treatment methods mainly include processes such as sedimentation, chlorination or activated slop. These processes work well for oxygen-binding substances, phosphates and nitrogen, but have not been further proven optimal for removing all pharmaceutical substances that end up in wastewater, as we can witness the increased impact of the pharmaceuticals on the living world in surface water. There are also some more advanced methods, including oxidation processes with ozonation, active carbon adsorption, membrane filtration, UV treatments, or biodegradation [5]. In the Netherlands, there is an initiative that will test new methods such as the REGAIN project that investigates the removal of medicine residues from treated sewage at the Garmerwolde treatment plant, and at the same time examines how this treated wastewater can serve as a source of industrial water [6]. Nevertheless, the above-mentioned processes cannot guarantee the complete removal of the pharmaceuticals ending up in water. In fact, during these processes transformation products are formed that might pose a bigger danger to the aquatic system [7]. This is a novel area that requires further research and attention in practice and in the education of pharmacy students and professionals. A recent study investigating samples of rivers in 104 countries found pharmaceutical residues in all but 2 countries [8] and the highest concentrations in countries where pharmaceuticals can be afforded by the population but sanitation is still limited.

Pharmaceutical residues impact the water world in various manners. For example, analgesics have been found to damage fish tissue and cause geno-/neurotoxicity to molluscs; hormones cause endocrine disruptions in frogs and induce sex changes in some fish; antipsychotics alter fish and invertebrates’ behavior; antibiotics may lead to antimicrobial resistance gene spread [9,10,11]. Moreover, when concentrations of pharmaceuticals increase along the food chain towards elevated levels in top predators in a trophic level, they are potentially consumed by humans as well [12]. In the Netherlands, a study found environmental risks for almost 20 different pharmaceutical residues, ranging from analgesics and hormones to anti-epileptics [1]. Studies indicated that the direct discharge of unused pharmaceuticals is limited to less than 10% compared to the human excretion in entering wastewater [13]. This implies that measures should be taken along the whole chain from medication production, prescription, use and, finally, the disposal of medication [14]. This is in line with the recommendations in the report of the Organization for Economic Co-operation and Development (OECD) which stresses the need for a collective approach of the stakeholders involved along the whole life cycle of medicines [9]. The current project has been initiated as part of the work of the Dutch network “Medicijnresten uit water Noord-Nederland”. This consortium is a great example of a broad cross-sector collaboration between the healthcare and the water sector in working together towards decreasing the effects of pharmaceuticals in water from various perspectives. See Box 1 for further details about this network, participating partner organizations and initiatives [15].

Box 1 **The Dutch network “Medicijnresten uit water Noord-Nederland”.**

**The network “Medicijnresten uit water Noord-Nederland” was established in 2019. Currently, this network brings 47 parties together. The broad cross-sector collaboration between the healthcare and water sector makes this network unique and also the largest in the Netherlands in combating the issue of pharmaceuticals in water.**

**Through ideas, initiatives and innovations, this cross-sectoral and multidisciplinary collaboration aims to use the strong position of the Northern Netherlands to become a leader in reducing medicines in water. A total of 19 parties from the network have developed 11 practice-oriented interventions during the past year and a half within the framework of an European Regional Development Funds (ERDF/REACT-EU) grant. These interventions support the problem from different perspectives, for example through education, product innovation and research. The education module “Reducing Pharmaceuticals in Water” is one of the 11 interventions, aimed at creating awareness among healthcare professionals.**

To be able to provide optimal advice and service to patients, healthcare professionals including pharmacists should be aware of the facts of how medicines impact the environment, particularly water, but so far this has not often been addressed in undergraduate education. Therefore, we developed a module for pharmacy students about environmental issues focusing on the pharmaceutical contamination of water. We have integrated this module in our pharmacy simulation game [16]. In brief, the pharmacy game simulates community pharmacy practice where students learn to make clinical and professional decisions in an authentic and safe environment that closely simulates real-world practice. Groups of students run their own simulated community pharmacy completing tasks such as setting up the mission and vision of their pharmacy, making a business and strategic plan, handling inspections and unexpected situations, processing prescriptions and counselling patients (played by actors). The whole course is managed using a web-based platform where the module “Reducing Pharmaceuticals in Water” was added as e-learning.

The aim of this study was to evaluate the effects of this module on students’ knowledge of pharmaceutical residues in water, describe students’ experiences in taking the module and explore their attitudes towards green pharmacy education in general.

## 2. Materials and Methods

### 2.1. Module Development and Description

Expert group meetings were held in the period between November 2022 and September 2023 to set up learning goals, a structure, content, a plan of implementation, use, and evaluation of the educational module “Reducing Pharmaceuticals in Water”. The group consisted of experts on the environment (*n* = 2), water technology (*n* = 1), higher education in the field of pharmacy (*n* = 2), pharmacy practice education (*n* = 2), and education program development (*n* = 1). This group of experts is part of a large consortium (Box 1) that combines various disciplines to work together towards mitigating the effects of the pharmaceutical residues found in water. The module was developed using the constructive alignment framework by Biggs and Tang [17]. One educational module is aligned when assessment and activities in the course support the learning objectives. In our module, we use Bloom’s taxonomy [18] to write down the learning objectives that reflect the highest cognitive level that is expected after finishing the course.

**Figure 1:**
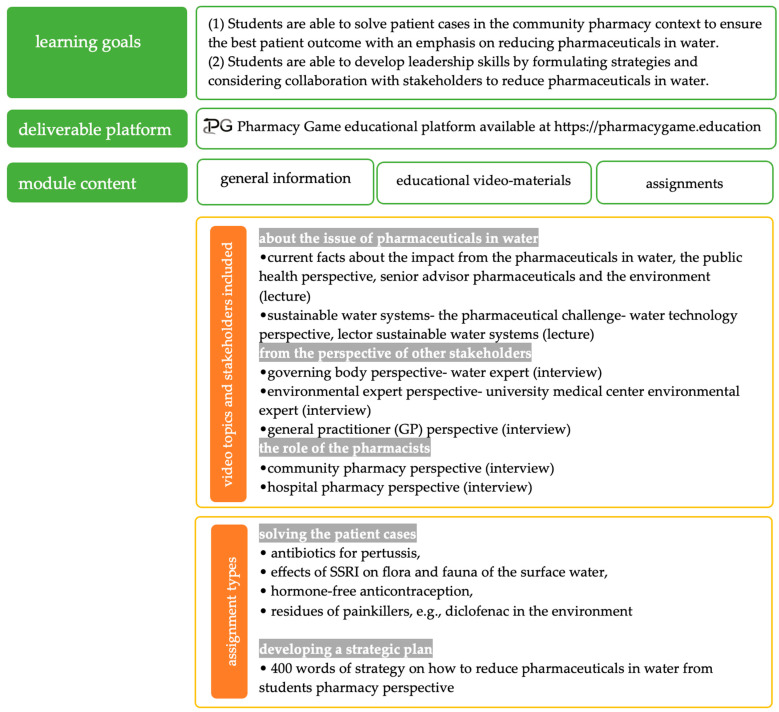
Structure of the educational module “Reducing Pharmaceuticals in Water”; PG—Pharmacy Game; SSRI—Selective serotonin reuptake inhibitors.

The module consisted of an e-learning component with practical tasks which was integrated into the innovative educational tool “The Pharmacy Game” [16], and taught to third year Master’s students in Pharmacy at the University of Groningen. The complete module structure is given in Figure 1.

The learning goals of the module were: (1) solve patient cases in the community pharmacy context to ensure the best patient outcome with an emphasis on water contamination and (2) become future healthcare leaders by developing strategies and considering collaboration with stakeholders to reduce drug contamination in water. To achieve these learning goals, various educational videos were created (total video length of 2 h). In the videos, experts gave lectures to provide a thorough knowledge base of the problem of pharmaceuticals in water to the students. Other videos were interviews with various stakeholders to provide perspectives on how to mitigate the problem of contamination. A particular focus was on the role of pharmacists in the processes by interviewing a community and a hospital pharmacist.

#### 2.1.1. Short Description of the Educational Video Materials

The lecture, representing the public health perspective, focuses on four domains: (1) Pharmaceuticals in the environment, explaining the occurrence of pharmaceuticals in most aquatic systems and the effects they have there; (2) Environmental risk assessment of pharmaceuticals, explaining what risk is, what a hazard is, how we determine these, and what happens afterward; (3) Legislative aspects, marketing authorization and the Dutch chain approach, explaining which legislative aspects are relevant to this issue, how the marketing authorization process works, and providing a focus on the Dutch chain approach to pharmaceuticals in the environment; and (4) Risk mitigation, explaining the risk mitigation measures and what we do when there is a risk of pharmaceuticals in the environment.

The following lecture, “Sustainable Water Systems—the Pharmaceutical Challenge”, brings water technology into perspective. Herewith, the lecturer discusses aspects of pharmaceuticals in domestic wastewater, their behavior during wastewater treatments, and the central perspective for improving water treatments. Pharmaceuticals can end up in surface water in various ways. For example, pharmaceuticals are prescribed, consumed, excreted via urine and feces, and transported with wastewater into the sewer system. This system brings the content into a sewage treatment plant. When the hydraulic capacity is overloaded, some wastewater ends up in the surface water before being treated because of combined sewer overflow, and it poses environmental risks and is a significant source of, for example, antibiotic-resistant genes. Of note, each pharmaceutical has a different removal rate from the sewage treatment plants, which can give pharmacists some idea about their potential impact on the environment or concretely on the water. It is based on the compound characteristics or ability of absorption and biodegradation. While sewage treatment plants are not made specifically to remove pharmaceuticals, some pharmaceuticals are effectively removed, such as paracetamol.

The perspective of a governing body is given through an interview video held with a representative of a local municipality. Their representative explains that the water systems’ quality, purification, and maintenance standards are the responsibility of the governing bodies or the municipalities. Besides maintaining their local initiatives, the municipalities are open to collaborative projects where stakeholders can learn from each other and contribute toward better protection of the pharmaceuticals in the water systems.

Another interview video included in the module was made with an environmental expert who shared information on current European and Dutch environmental initiatives that address pharmaceuticals in the waters. The Green Deal in Europe is an integrated plan that covers all large sustainable challenges in the future. At the same time, the Green Deal in the Netherlands represents agreements between the government and individual sectors or regions. It recognizes the need for collaboration between hospitals, other healthcare sectors, and organizations to achieve the best patient care and protect the environment. An example of collaborative practices is the project where the concentration of pharmaceuticals is measured in wastewater to realize the potential impact and ensure undertaking future preventive measurements. This unites three stakeholders: the hospital (sample provider), the municipality (analyses and policy), and a private company (performing the testing and the analyses).

The following video interview is carried out with a general practitioner (GP) as the prescriber of medication in primary care. In the interview, the GP reflects on whether the prescribers are aware of the pharmaceutical impact, what they are doing to prevent pharmaceuticals from ending up in the water, and what can be done in the future. It is also identified that GPs should become more familiar with this issue, start with initiatives in their practices, and try to disseminate those in the regional and national meetings for exchanging best practices. According to the interviewed GP, more attention should be given to the need for prescribing and following up on the proper utilization of and need for further treatments.

Furthermore, a community pharmacist is interviewed, as the first line of care in direct contact with the patient. They therefore represent crucial health workers for protecting the environment and water from medical residues. In the interview, the community pharmacy specialist shares practical experiences. The community pharmacists emphasized the increased interest in the topic among their colleagues, but also the need for more specific information. That resulted in a focus on collecting unused medicines in the community pharmacy, as current practices and insurance policies in the community pharmacy are areas for improvement.

The final interview is carried out with a hospital pharmacist as these are essential in patient care and medication management within hospitals. The highly toxic residues disposed of in medical centers and hospitals imply potential environmental hazards and risks. The hospital pharmacist-clinical pharmacologist-epidemiologist shares experiences gained over the years and highlights recent examples of thinking green in hospital settings. This video interview brings into perspective some initiatives and models from the hospital, including the Green Deal initiative, reducing unnecessary medication use, reusing medication, preventing inappropriate disposal, and using medicines with a lower impact on the surface water.

#### 2.1.2. Short Description of the Assignments

After watching the videos, students had to complete two assignments which were integrated in the pharmacy simulation game course [16]. The two assignments of the new module created to address the learning goals were:

(1) Solving patient cases (*N* = 4): (1) antibiotics for pertussis, (2) selective serotonin reuptake inhibitors (SSRI) effects on flora and fauna in the surface water, (3) hormone-free contraception, and (4) residues of painkillers, e.g., diclofenac in the environment. The medicines used in the cases were chosen from published lists [2,19]. An illustration of a case is given in the Supplementary Materials. Students’ advice to the simulated patients was assessed (content and communication skills as previously established and put into practice within the Pharmacy Game course at the University of Groningen [16]). This was an individual assignment.
(2) Writing a strategic plan on how to reduce pharmaceuticals in water from their pharmacy perspective: Each group of students had to prepare a strategic plan of a maximum of 400 words comprising ideas about reducing pharmaceuticals in the water in line with the mission and vision of their pharmacy. In the plan, they had to consider the stakeholders, the activities, and the patient impact. This assignment was assessed based on a rubric assessment that is standardized to allow answers on the assigned tasks and gives them freedom to be creative in their written feedback. Four areas were assessed: (1) Inclusion of innovation element; (2) If the strategic plan is in line with mission and vision of the pharmacy; (3) Which stakeholders are included and how are they involved; (4) Patient inclusion. Each of these areas brought from −10 to +10 points, to total a maximum of 40 points [16].

### 2.2. Study Design

This was a mixed-methods study including quantitative measurements before and after students took the module (intervention group) and a single measurement in a control group, which did not receive the module. Ethical clearance was not requested as outlined in a corresponding statement given in the Supplementary Materials.

#### 2.2.1. Participants

The study population consisted of students enrolled in the Master of Science in Pharmacy at the University of Groningen, the Netherlands. We recruited students participating in the pharmacy simulation game [16] in the third year of the Master. The intervention group (*N* = 29), was composed of students taking the course in May–June 2023. The students taking this course in January–February 2023 served as a control group (*N* = 36).

#### 2.2.2. Measurement Instruments

We created *a questionnaire to assess students’ knowledge*. The questionnaire consisted of: (1) Introduction/consent for participation, (2) General information about the respondent/demographic information (open and closed questions), and (3) Knowledge about reducing pharmaceuticals in water (22 multiple choice questions). Questions were developed by the expert group responsible for the module development, described in Section 2.1, and in line with the current directions for developing high-quality multiple choice questions [20].

The questionnaire can be found in Supplementary Materials (Table S1). The questionnaire took around 15 min to complete. By design, participants were not identifiable and consented to participate in this study.

We used *a course evaluation questionnaire* [21], which was further adjusted by two researchers (T.F. and K.T) to describe students’ experiences in taking the module. The adjustments deemed necessary for capturing the novel aspects of the developed module included the design of the module (e-module), its specific (video) content and the specially designed assignments for this module, the patient cases and the strategic plan. A five-level Likert-scale [22] was used to collect students’ opinions, from “completely disagree” to “completely agree” on the different parts of the module. An open question was added at the end of this questionnaire to allow students to provide additional remarks. The complete evaluation form is given in the Supplementary Materials (Table S2).

*The attitudes of the pharmacy students towards green pharmacy education* were explored using the questionnaire developed in Finland by Siven et al., 2020 [23]. The authors gave permission to use their questionnaire consisting of four closed and one open question.

#### 2.2.3. Data Collection

The students’ knowledge (22 multiple choice questions, Supplementary Materials, Table S1), based on the video materials described in Section 2.1.1, was assessed twice in the intervention group, first in May 2023 and second in June 2023, which was after completing the module. The knowledge was also assessed in the control group, in one measurement (February 2023). These students did not receive the module. The questionnaire on the attitudes of the pharmacy students towards green pharmacy education was also completed twice by the intervention group and once by the control group. The course experience questionnaire was completed only once by the students in the intervention group after completing the module. The questionnaires were completed on paper to avoid using the internet to search for correct answers. Overall, data collection was conducted from February 2023 to June 2023.

#### 2.2.4. Data Analysis

Demographic characteristics were assessed descriptively. To determine students’ knowledge, the sum score was calculated for each respondent by adding the scores of the different questions of the questionnaire (1 = correct answer; 0 = incorrect answer); the total possible score was therefore 22. A *t*-test was performed to compare the mean sum score of the first and second measurement for the intervention group. If assumptions for a t-test were not met, i.e., normal distribution and similar variance of the two measurements [24], a Mann–Whitney U-test was performed. A significance level of α = 0.050 was used for both the *t*-test and the Mann–Whitney U-test [24]. All other data were presented using descriptive statistics.

## 3. Results

In total, 29 students followed the module. Students showed the best performance related to the case “effects of SSRI on flora and fauna of the surface water” and worst related to the “antibiotics for pertussis” case (Supplementary materials, Table S3). An example of a strategic plan developed by one group of students is given in Supplementary Materials (Table S4). A total of 36 students were recruited as the control group. Demographic information of the responders to the questionnaires is given in Table 1.

### 3.1. Effects of the Module on Students’ Knowledge of Pharmaceutical Residues in Water

The knowledge score of students in the intervention group increased significantly from 9.3 to 12.9 (*p* < 0.001). The knowledge score of the students in the control group was 8.9 out of a total maximum score of 22.

### 3.2. Students’ Experiences in Taking the Module

**Figure 2:**
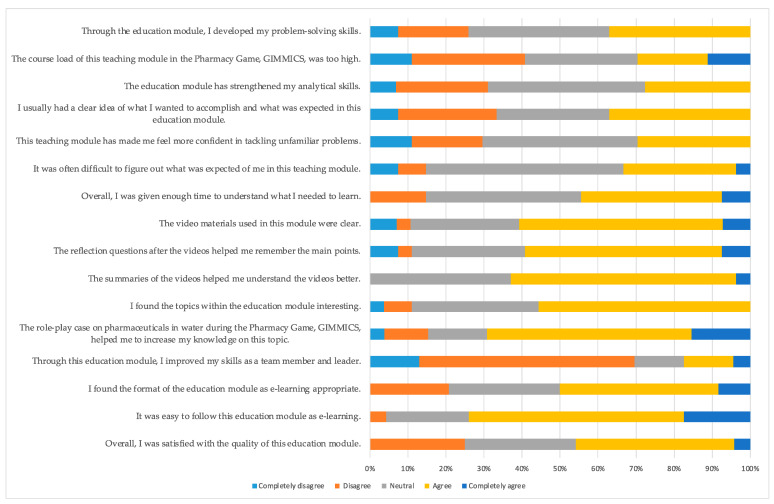
Students’ evaluation of the education module “Reducing Pharmaceuticals in Water”. GIMMICS^®^ (Groningen Institute Model for Management in Care Services).

A total of 27 out of 29 students completed the course experience questionnaire (93%). The majority of the students found the topic and the patient cases interesting. The module was easy to follow in its given form. According to the students, this module is less likely to improve their skills as team members and leaders, and about a third found that the work load was too high. Notably, a considerable proportion of students neither agreed nor disagreed with many statements. The complete evaluation results are given in Figure 2.

A total of 11 out of 27 students answered the open question of the course experience questionnaire (40%). The majority of the students expressed the necessity to add a lecture before the module that will bring more concept to the topic. A few students found some videos too long, while some indicated preferences for the videos with practical examples within a pharmacy or general practitioner practice. The complete descriptions of these results are given in Supplementary Materials (Table S5).

### 3.3. Pharmacy Students’ Attitudes towards Green Pharmacy Education

The majority of the control group (69.4%) and the first measurement of the intervention group (55.5%) stated that green pharmacy topics were insufficiently included in the education. This percentage was reduced to 18.5% in the intervention group after following the module (Table 2). Furthermore, the majority of pharmacy students were in favour of integrating the topic into selected courses (Figure S1a,b); they also stated that the studies must contain environmental issues (Figure S1c), and the majority (Figure S1d) consider that drug developers and manufacturers should consider sustainability and environmental viewpoints.

## 4. Discussion

### 4.1. Main Findings

We developed a module to teach pharmacy students about pharmaceutical residues in water. The module consisted of educational videos and assignments which were integrated into the Pharmacy Game educational platform. Students were significantly more knowledgeable about the topic after completing the module. Students found the deliverable form (e-learning) and the patient cases the most noteworthy part of this module. Students wished to have more green pharmacy content in their undergraduate education.

### 4.2. Interpretation

There is a need for implementing environmental sustainability and addressing the ecological impact of pharmaceuticals in pharmacy education [23,25]. This is also stated by the EU Pharmaceutical Committee, who mandated an ad hoc working group to ”Explore, in cooperation with relevant stakeholders, how environmental aspects could become part of medical training and professional development programs” [26]. Some examples of how this has been addressed include learning session within the clinical pharmacology subject in the Faculty of Pharmacy at the University of Helsinki, where students discussed a patient case at the beginning and end of the course, a module in the P-Scribe system [27] of Maastricht University, and a module for continuous education of doctors and pharmacies [14]. More extended education has been implemented in Spain, where the University offers an 8-month postgraduate course on pharmaceutical pollution, intended for healthcare professionals [28]. Recently, a unique international Master in sustainable drug discovery has been set up [29]. These examples comprise short lectures to complete programs, showing that depending on the target audience, the level of detail in a course may differ. Assessment methods were also diverse, including discussion or a knowledge test about a patient’s case. We have created a short module which teaches knowledge and enhances skills and attitudes of the students focusing on pharmaceuticals in water; this is suitable for pharmacy undergraduate students, but may also be used for other groups such as practicing pharmacists or other health professionals.

A recent study investigating students’ knowledge of the issue of pharmaceuticals in the environment reflected relatively poor knowledge among the 186 tested pharmacy students at the University of the Basque Country [30]. Their study used 13 questions with the possibility of yes/no, or scaled answers. Three of the questions overlap with the ones we used in our research, two discussing the Environmental Risk Assessment for the authorization of new medicines in Europe [31], and one concerning the impact of antibiotics. We tested the knowledge with 22 multiple choice questions based on the video materials of our module. The students’ knowledge was lower before than after following the module. Despite the differences between the Spanish and our study, both highlight the need to provide education on the environmental aspects of pharmaceuticals. Lack of knowledge was also demonstrated in a Chinese study looking into knowledge of ecopharmacovigilance in practicing pharmacists, which showed a knowledge score of 3.85 out of 10 [32]. An Australian (interview-based) study in a hospital pharmacy workspace showed similar results [33]. Moreover, an international survey study conducted among community and hospital pharmacists to explore the pharmacists’ activities for reducing medication waste showed higher scores for the importance of such activities rather than their visibility in practice [34]. All of the latter studies indicate the need for improving the knowledge among pharmacists in practice.

The results of the course experience questionnaire show the elements which were appreciated by the students and the elements which can be improved. The students liked the videos which were available online through the educational platform. They also appreciated the student engagement tasks with the reflection questions and summaries after the videos. However, students found some videos too long (45 min) and less interesting than others. Therefore, we will update some of the video lectures accordingly. The four patient cases were much appreciated by the students. Course teachers saw a variation in student’s performance when playing the case and in the strategies that students wrote. Nevertheless, the aspect of applying the knowledge by playing the patient cases and writing the strategy is an important part of the module and we will keep these two activities. We initiated two changes. First, an introductory lecture will be organized to guide the students through the module content and assignments, and how those reflect the learning goals. Second, a group session will be held to reflect on each other’s roles and participation in this module. Finally, we will continue to investigate students’ experience using the questionnaire.

We used the same questionnaire as Sivén et al. [23] to investigate our students’ attitudes about green pharmacy education in general. Slightly more than half of our students indicated that environmental aspects are insufficiently addressed in the current pharmacy curriculum. This is in between 75% and 33% reported by Sivén et al. in their studies in 2015 and 2019, respectively, showing differences in the degree to which these topics have been integrated in the Finnish curriculum. In line with the Finnish students, our students prefer to integrate it in selected courses rather than introduce separate courses on the topic. To this end, the results give guidance on the next steps to integrate the subject in our curriculum further.

### 4.3. Strengths and Limitations

Our study adds to the growing body of literature on how to integrate environmental aspects in pharmacy education in describing the development and evaluation of a complete module that addresses the issue of pharmaceuticals in water. Creating this module involving different stakeholders is unique as it combined forces from a large consortium (Box 1). It was developed using state-of-the-art didactical methods such co-alignment and co-creation with stakeholders [17]. We were able to integrate the module in an existing course using a unique teaching method combining simulation and gaming [16,35].

Module evaluation results should be treated with caution as not all students watched the video lectures, because they gave neutral answers to the questions related to the videos. Another limitation is the study design. We measured knowledge before and after in the intervention group, but were only able to obtain knowledge scores once in the control group. In the absence of validated measurement instruments to assess knowledge about the environmental aspects of pharmaceuticals, we developed our own knowledge questionnaire, based on the educational materials. Our study provides important information on the further development of our module. It also informs future endeavors of more rigorous evaluations of education.

### 4.4. Future Implications for Education and Research

We implemented the module in the frame of the existing compulsory course, the Pharmacy Game [16], but the module is comprehensive enough (about one European Credit Point) to be used as an independent module for pharmacy students. Thereby the module is suitable to be integrated in other curricula. Possibly only small adaptations are needed to make this module fit for other countries and health care systems. Research on the international level may contribute towards improving the module content, but also towards greater awareness outside Dutch borders. Furthermore, the module is potentially suitable for postgraduate education for pharmacists. The module can be a part of the “Planetary Health” curriculum [36]. How to connect the module with other parts of green pharmacy education to achieve a complete and comprehensive education for undergraduate pharmacy students should be further explored.

## 5. Conclusions

“Reducing Pharmaceuticals in Water” is a comprehensive module for pharmacy students to improve knowledge and increase awareness about the environmental impact of pharmaceuticals. The survey research further informed us about the need to address the issue of pharmaceuticals in water more profoundly in pharmacy education. Interventions like the educational module we developed can help in that matter. Increasing knowledge and awareness among healthcare professionals, accompanied by concrete actions from multiple stakeholders, is a promising strategy to mitigate the impact of pharmaceuticals on water and the environment in the future.

## Figures and Tables

**Table 1:** Demographic information of responders.

Characteristics	Control Group Total *N* = 36	Intervention Group Total *N* = 29
**Age in years, mean (SD; range)**	24.6 (2.1; 12)	24.9 (1.5; 7)
**Gender**	***N*, %**	***N*, %**
Female	26, 72.2%	22, 75.9%
Male	9, 25.0%	7, 24.1%
Prefer not to disclose	0, 0.0%	0, 0.0%
Other	1, 2.8%	0, 0.0%

**Table 2:** Opinions on the question if the green principles and environmental viewpoints are presented in pharmacy education.

	Control Group Measurement Total *N* = 36	Intervention Group	
		First Measurement	Second Measurement
		Total *N* = 29	Total *N* = 27
**Responses**	***N,* %**	***N,* %**	***N,* %**
**Adequately**	1, 2.8%	0, 0.0%	3, 11.1%
**To some extent**	10, 27.8%	13, 44.8%	19, 70.4%
**Not enough**	25, 69.4%	16, 55.2%	5, 18.5%
**Total**	36, 100%	29, 100%	27, 100%

## Data Availability

The original contributions presented in the study are included in the article/supplementary material, further inquiries can be directed to the corresponding author/s.

## Supplementary Materials

The following supporting information can be downloaded at: https://www.mdpi.com/article/10.3390/pharmacy12010028/s1, Table S1: Questionnaire; Table S2: Module evaluation form; Table S3: Students’ performance of the pharmaceuticals in water related patient cases; Table S4. Example of strategic plan developed by one of the teams, participating in the Pharmacy Game course at the University of Groningen May–June 2023; Table S5: Student responses in regard to the module improvements through the additional open question; Figure S1a: Students’ responses to question *How should the green principles and environmental viewpoints regarding medicines be presented in studies? (Multiple choices are possible*; 65 responders from the baseline measurements of the control and intervention group); Figure S1b: Students’ responses to question *Which courses should contain green pharmacy contents (Open question*; 65 responders from the baseline measurements of the control and intervention group); Figure S1c: Students’ responses to question *In the following, you will find selected statements. Please, select the ones that you consider relevant in curriculum reform and future progress towards green pharmacy practices in education. (Multiple choices are possible*; 65 responders from the baseline measurements of the control and intervention group); Figure S1d: Students’ responses to question *Which are the stakeholders that should consider sustainability and environmental viewpoints regarding medicines?* (*Multiple choices are possible*; 65 responders from the baseline measurements of the control and intervention group); A short description of the patient case “*Hormone-free contraception*” used in the module; and Ethical clearance statement.
