# Supplementary material for: Reducing Pharmaceuticals in Water, a New Module Integrated in the Pharmacy Game: Evaluating the Module’s Effects on Students’ Knowledge and Attitudes
Source: Pharmacy (Basel). 2024 Feb 6;12(1):28. doi: 10.3390/pharmacy12010028 (PMC10892046; doi:10.3390/pharmacy12010028)
Supplement: Supplementary file 1 [file pharmacy-12-00028-s001.zip › pharmacy-2777673-supplementary.pdf]

Supplementary materials:

Table S1. Questionnaire

Introduction/ consent for participation:

Do you consent to the use of this information for further education and research purposes?
a) YES<br>b) NO
Date when the question was answered (dd/mm/yyyy)

General information about the responder/ demographic information:

In which year did you start your BACHELOR pharmacy study?
In which year did you start your MASTER pharmacy study?
What is your current age?
What is your gender?
a) Female<br>b) Male<br>c) Prefer not to disclose<br>d) Other (please specify) ________________
Did you follow the educational module: *"Reducing pharmaceuticals in water"*
a) YES<br>b) NO

Questionnaire: Awareness and knowledge about reducing pharmaceuticals in water

(One answer possible)

What is the Green Deal?

a) Initiative for working together towards sustainable care
b) Environmental organization about sustainable care
c) Energy-saving instrument for sustainable care
d) Political party for sustainable care

What is the goal for decreasing the CO2 emission by 2030, compared to 2018:

a) 55% less direct CO2 emission
b) Climate neutral
c) 30% less direct CO2 emission
d) 10% less direct CO2 emission

Using raw materials responsibly and therefore achieving a cleaner and healthier world for the current and future generations is a process known as:

a) Circularity
b) Raw material movement
c) Purifying
d) Environmental activism

What is the most relevant effect of pharmaceutical residues in water on aquatic ecosystems?

a) Acute mortality
b) Effect on oxygen levels
c) Effect on reproduction
d) Acute disease

Decreasing the amount of pharmaceutical residues in water impacts:

a) Global warming
b) Antibiotic resistance
c) Depression
d) Chronic diseases

Which element of the drug discovery development process is in the focus of the publication "GREENER Pharmaceuticals for More Sustainable Healthcare" ?

a) The medical dossier
b) The active pharmaceutical ingredient
c) The GMP standards
d) The health policy

The Environmental Risk Assessment in the legislation for human medicines is used for:

a) Establishing the risk to the environment due to the *use* of the product
b) Establishing the risk to the environment due to the *production* of the product
c) Establishing the risk to the environment due to the *discarding* of the product
d) Establishing the risk to the environment due to the *nature* of the product

An existing risk, reported in Environmental Risk Assessment, can be a reason to refuse marketing authorization for:

a) Medical devices
b) Food supplements
c) Human medicines
d) Veterinary medicines

The risk assessment in Environmental Risk Assessment for pharmaceuticals depends on:

a) Effect and exposure
b) Prediction and measurement
c) Observation and elimination
d) Concentration and mutations

Risk characterization in Environmental Risk Assessment is the relation (ratio) between:

a) Predicted *effect* concentration and predicted *non-effect* concentration
b) Predicted *eliminated* concentration and predicted *non-eliminated* concentration
c) Observed exposure and non-exposure
d) Observed effects in the lab and observed effects in the field

The effect assessment for the Environmental Risk Assessment of human-pharmaceuticals is assessed based on effects on three trophic levels including:

a) daphnia- fish-reptiles
b) algae- daphnia- fish
c) water plants- daphnia- fish
d) water plants- daphnia- reptiles

The Dutch healthcare sector is responsible for:

a) 10% of waste and the use of 40% of all raw materials in the Netherlands
b) 4% of waste and the use of 13% of all raw materials in the Netherlands
c) 1% of waste and the use of 8% of all raw materials in the Netherlands
d) 15 % of waste and the use of 20% of all raw materials in the Netherlands

Which percentage of *pharmaceutically active compounds* are removed (on average) through existing wastewater treatment plants?

a) 10%
b) 30%
c) 65%
d) 90%

How much medicine waste is estimated to be emitted into surface waters every year in the Netherlands?

a) 50 ton/year
b) 100 ton/year
c) 200 ton/year
d) 400 ton/year

It is expected that the costs of domestic wastewater treatment will increase when additional techniques are installed for the advanced removal of pharmaceuticals from domestic wastewater. What is the estimated increase in costs (per inhabitant) when additional treatment techniques are implemented?

a) 5-10 euro extra per inhabitant per year
b) 10-25 euro extra per inhabitant per year
c) 60-70 euro extra per inhabitant per year
d) 100-110 euro extra per inhabitant per year

Which of the following *pharmaceutically active compounds is hardly* removed in *wastewater treatment plants,* and as a consequence, is generally detected in surface waters?

a) Ibuprofen
b) Carbamazepine
c) Trimethoprim
d) Sulfamethoxazole

Discharge of untreated wastewater via Combined Sewer Overflows are a major source of:

a) Antibiotics
b) Antibiotics-resistance genes
c) Pharmaceutical wastage in general
d) Artificial sweeteners

Pharmaceutical residues from painkillers in water may cause:

a) Histopathology in fish
b) Antimicrobial resistance
c) Behavioral changes in vertebrates
d) Less growth of algae

The presence of antidepressants in surface water may impact:

a) Certain fish and mollusks
b) Algae
c) Water flora
d) The water density

How do the Selective Serotonin Reuptake Inhibitors impact the organisms in surface water?

a) Induce alterations in genetic transcription, deficiency in reproduction and motility
b) Induce neurodegeneration and mortality
c) Induce increased sensitivity of the membranes and surface tissues
d) Induce deficiency in responsiveness in sensory organs

Medicines residues from contraceptive pills in water induce fish becoming:

a) Bigger
b) Feminine
c) Calmer
d) Weaker

What kind of contraception could pharmacists/ doctors recommend which have the least impact on water pollution?

a) Oral contraceptives
b) Intrauterine device
c) Contraceptive implants
d) Condoms

Table S2. Module evaluation form

This evaluation form is given in Dutch language as it was used among the students. We choose for the Dutch language so that the student can better reflect their opinions in regard to this education module.

Het doel van deze vragen is om de percepties van studenten over de onderwijsmodule* "Reducing pharmaceuticals in water" te verzamelen.

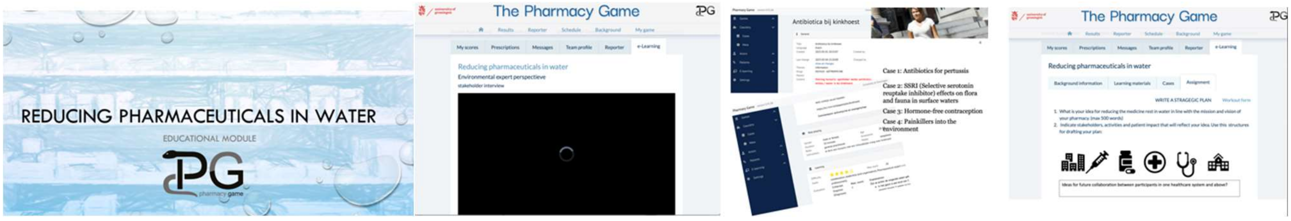

*Met onderwijsmodule bedoelen we: 1) de video's met reflectievragen, 2) de vier patientcasussen (Antibiotica bij kinkhoest; Effecten van SSRI op flora en fauna in oppervlaktewateren; Hormoonvrije anticonceptie and Restanten van pijnstiller (diclofenac) in het milieu) en 3) het strategische plan dat online ingevuld moest worden.

	Vraag	Helemaal oneens	Oneens	Neutraal	Eens	Helemaal eens
1	Door de onderwijsmodule heb ik mijn probleem-oplosvaardigheden ontwikkeld.					
2	De studielast van deze onderwijsmodule in the Pharmacy Game, GIMMICS was te hoog.					
3	De onderwijsmodule heeft mijn analytische vaardigheden versterkt.					
4	Ik had meestal een duidelijk beeld van datgene wat ik wilde bereiken en wat er van me verwacht werd bij deze onderwijsmodule.					
5	Deze onderwijsmodule heeft ertoe geleid dat ik mij zekerder voel in het aanpakken van onbekende problemen.					
6	Het was vaak moeilijk om te ontdekken wat er in deze onderwijsmodule van me verwacht werd.					
7	Over het algemeen kreeg ik genoeg tijd om de dingen die ik moest leren, te begrijpen.					
8	De videomaterialen die werden gebruikt in deze module waren duidelijk.					
9	De reflectie vragen na de video's waren behulpzaam bij het onthouden van de belangrijkste punten.					
10	De samenvattingen van de video's hebben mij geholpen om de video beter te begrijpen.					
11	De onderwerpen binnen de onderwijsmodule vond ik interessant.					
12	De casussen over medicijnresten in water tijdens Gimmics hebben bijgedragen aan het vergroten van mijn kennis over dit onderwerp.					
13	Door deze onderwijsmodule heb ik mijn vaardigheden als teamlid en leider verbeterd.					
14	Ik vond de opzet van de onderwijsmodule als e-learning passend.					
15	Het was makkelijk om deze onderwijsmodule als e-learning te volgen.					
16	Over het algemeen was ik tevreden over de kwaliteit van deze onderwijsmodule.					
17	Andere opmerkingen?					

Table S3. Students' performance of the pharmaceuticals in water related patient cases

	Case 1		Case 2		Case 3		Case 4		Total number of points per pharmacy team per criteria
	content	communication	content	communication	content	communication	content	communication	
**Pharmacy team 1**	-9		14		13		8		26
		1		12		17		14	44
**Total number points per case**	**-8**		**26**		**30**		**22**		
**Pharmacy team 2**	-6		7		8		2		11
		13		18		15		5	51
**Total number points per case**	**7**		**25**		**23**		**7**		
**Pharmacy team 3**	14		17		7		9		47
		14		16		15		18	63
**Total number points per case**	**28**		**33**		**22**		**17**		
**Pharmacy team 4**	-10		0		4		-5		-11
		0		6		11		1	18
**Total number points per case**	**-10**		**6**		**15**		**-4**		
**Pharmacy team 5**	-16		13		4		1		2
		0		18		13		4	35
**Total number points per case**	**-16**		**31**		**17**		**5**		

CASE 1: antibiotics for pertussis, CASE 2: effects of SSRI on flora and fauna of the surface water, CASE 3: hormone-free anticontraception, and CASE 4: residues of painkillers e.g., diclofenac in the environment.

The table gives a preview of the points (from -40 to +40 per case) students gained while playing the 4 cases. Each case was scored for 2 criteria, content (white fields) and communication (green fields). Students showed better communication skills than knowledge around the content of the case in general. The total number per case is given in the blue fields, showing that the case discussing the issue of antibiotics for pertussis and its impact on the waters was the most difficult for the students to solve.

Table S4. Example of strategic plan developed by one of the teams, participating in the Pharmacy Game course at the University of Groningen May-June 2023.

Pharmacy Team: "Apotheek Goedbloed"
Currently, our pharmacy is already working on reducing pharmaceuticals in two ways. The first project we are working on was initially not set up to reduce pharmaceuticals in water, but has the potential to help with environmental issues. Secondly, our pharmacy has set up a project to encourage patients to help with the reduction of pharmaceutical waste. Our pharmacy has the goal to create even more projects to stimulate the reduction of pharmaceutical waste, one of these will be described in this document.<br>*A part of our care-project "medicijn-maat", is performing medication reviews. With these reviews, it is possible to find out if our patients can use less drugs than they do now. With this reduction, the amount of waste can also be reduced. But it also is in line with our mission and vision, because we can focus more on single patients and thus enhance individual care. For instance, they can talk about their therapy-related problems and therapy compliance. By helping out patients with enhancing their compliance, less drugs are being wasted and thus less pharmaceuticals reach the water.*<br>*Our second project that's running, is the project "blossom the city". With this project, we reward patients for returning their medicinal waste to the pharmacy, by giving them flower seeds in return. These flower seeds can flourish the town or their homes, but also help in creating a cleaner environment. Above that, working with flowers outside is good for mental health, stress reduction and vitamin D. We see a weekly increase in returns of waste from patients, so we have good hopes that in the future*

returning medication waste has become a normal procedure for every patient. When patient return their pharmaceutical waste to our pharmacy, we are able to discard it in a responsible way.
For the future, we also want to explore possibilities of using more sustainable, recyclable or biodegradable packaging. For this, we need the pharmaceutical industry, manufacturers and other suppliers to come together and discuss possibilities. For this plan to succeed, we also need the patients to return all their medication waste to the pharmacy. We hope that with our project "blossom the city", we can raise enough awareness to ensure that every patient returns their packaging to the pharmacy so that it can be recycled.

Table S5. Student responses in regard to the module improvements through the additional open question

	**student responses**
**Student 1**	Some videos were too long. We had no time during the course to watch them all with full attention. Maybe some can be shortened in the future.
**Student 2**	It is nice and educational to make a PAO activity so that the topic is easier in the spotlight. I would have also liked it if more attention was given to the different patient- types rather than different cases.
**Student 3**	I would preferer to also have a PAO activity to introduce to the topic. An information about which substances are most polluting for the environment and how to interpret this as pharmacists should be nice to have available within the module.
**Student 4**	It would have been useful to add a PAO (post-academic education) activity at the beginning of this module.
**Student 5**	The number of cases on this topic may be reduced to 2 instead of 4.
**Student 6**	I would have love to have a lecture about this topic, as there was too little time to go through all the module materials which we did in between other activities, therefore I got only a general perspective. The cases have helped to go deeper in the topic.
**Student 7**	Maybe presenting the topics in the videos as a different PAOs would have been more interactive. We really did not have time to pay attention on all video materials. The information provided in the 4 cases will definitely remain in my head.
**Student 8**	With all the activities during the Pharmacy Game, my motivation to follow this module was low. For me would have been useful if I could play the videos faster as some of the speakers talked too slow.
**Student 9**	The topic is important but some of the movies were less interesting than other. I do not understand the question about how my leadership skills were improved with this module.
**Student 10**	Not all videos were equally interesting. To me, the ones from the general practitioner and hospital pharmacists' perspective were very relevant including interesting and concrete information I will remember. The patient cases were nice and challenging!

**Student 11**	It was a bit too much. Only the e-learning + one or two cases would have been enough. There is not enough information to find around this topic when solving a case.

PAO-post-academic education is an activity that we offer in form of lecture to the students to introduce them with different actual topic. This activity simulates the educational upgrades that pharmacists do after receiving their licenses and need to collets points to keep their license.

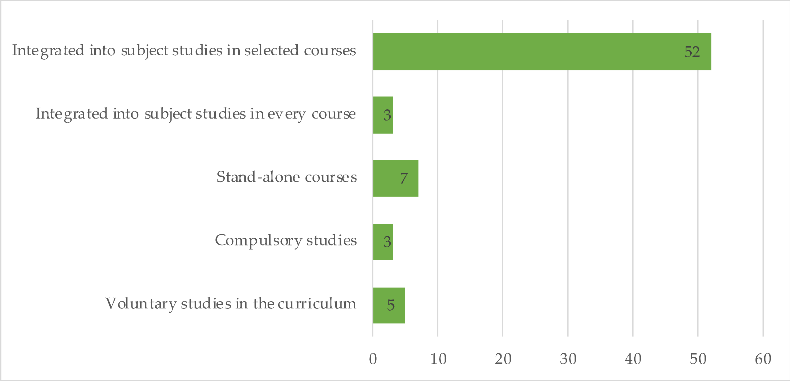

Figure S1-a Students' responses to question *How should the green principles and environmental viewpoints regarding medicines be presented in studies? (Multiple choices are possible*; 65 responders from the baseline measurements of the control and intervention group*)*

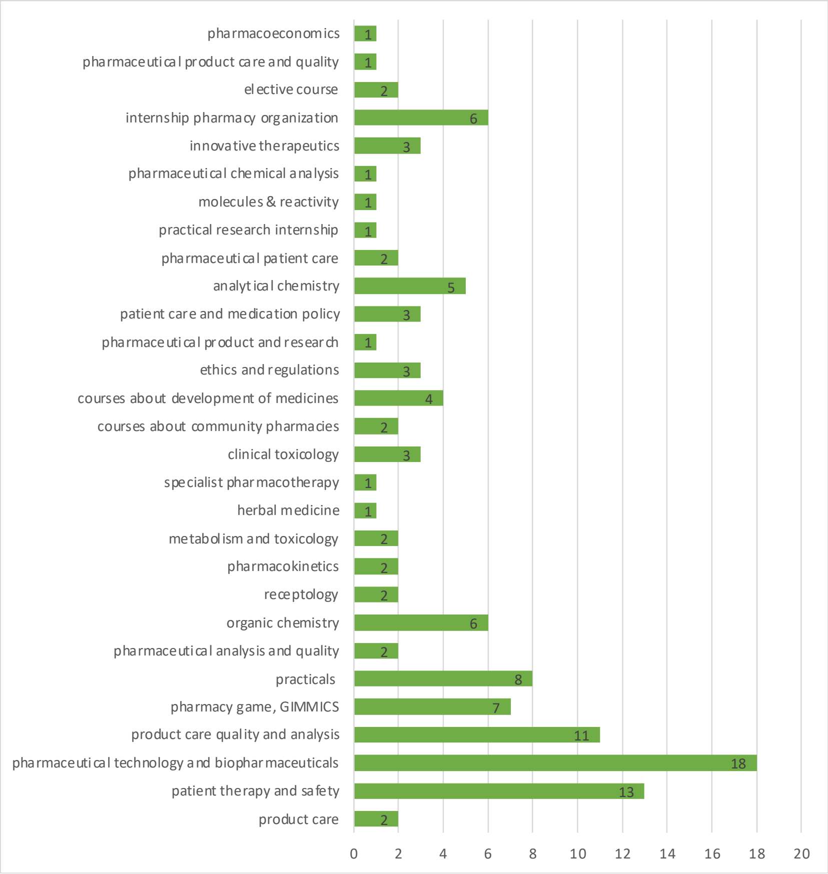

Figure S1-b Students' responses to question *Which courses should contain green pharmacy contents (Open question;* 65 responders from the baseline measurements of the control and intervention group*)*

This figure indicates student choices, where the module content can complement the existing pharmacy program (https://www.rug.nl/masters/medical-pharmaceutical-sciences/?lang=en). The figure shows a variation of subjects distributed through the bachelor and master of pharmacy program at the university of Groningen.

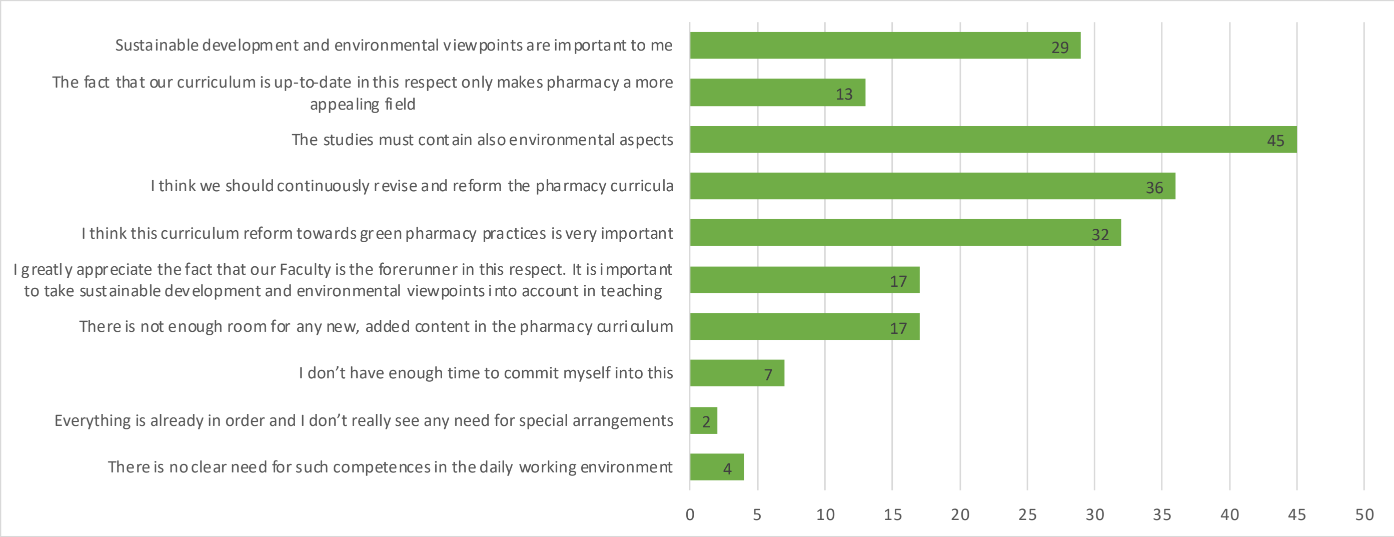

Figure S1-c Students' responses to question I*n the following, you will find selected statements. Please, select the ones that you consider relevant in curriculum reform and future progress towards green pharmacy practices in education. (Multiple choices are possible*; 65 responders from the baseline measurements of the control and intervention group*)*

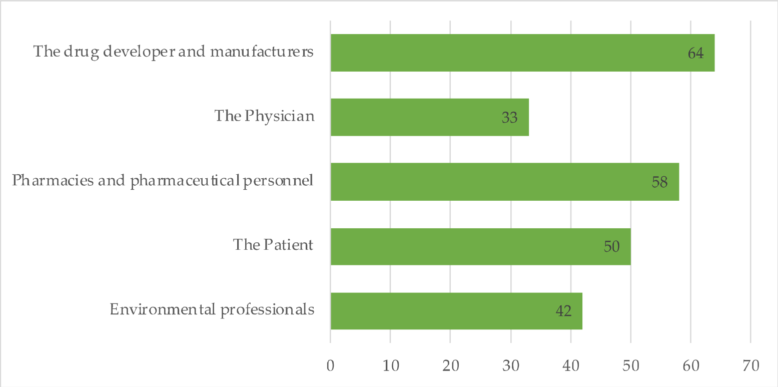

Figure S1-d Students' responses to question *Which are the stakeholders that should consider sustainability and environmental viewpoints regarding medicines? (Multiple choices are possible*; 65 responders from the baseline measurements of the control and intervention group*)*

A short description of the patient cases "*Hormone-free contraception*" used in the module

A woman between 30-36 years old, mother of two young girls, Emily and Julia, uses the contraceptive pill Microgynon 30 mcg. She is very concerned about the pollution in the environment and especially the one in water. She reads in the newspaper that the water contains more and more female hormones and how harmful this is to the fish. Therefore, she is looking for a contraceptive method

that does not contain hormones. Currently, she uses a birth control pill (Microgynon 30mcg). Preferably, she would like to have a contraception that would not harm the environment.
Care question: hormone-free birth control.
- What aspects of contraception are essential to the woman/couple?
- Sterilization is not an option.
- No desire for children in the next 2-3 years, probably after that.
- Previous use of contraception: Currently, the patient uses the birth control pill Microgynon 30 mcg (takes a pill every day for three weeks, then a stop week).
- From the pharmacist, the patient likes to hear what types of contraception are available and which of them are hormone-free.
- Ultimately, the patient chooses to have a copper IUD placed, and she requests information about it.
This patient experiences the side effects of the birth control pill that she is currently using, such as headaches and sometimes vaginal bleeding outside the stop week (also called "spotting"). She wonders what side effects she can expect with the copper IUD or hormone IUD Mirena.

Supplementary materials: Ethical clearance statement

This research was conducted in compliance with all relevant national and international laws, rules and agreements. The research does not falls under the Medical Research Involving Human Participants Act (WMO) or any other applicable laws and regulations that impose conditions on the research, such as the Regulation on the protection of natural persons with regard to the processing of personal data and on the free movement of such data, and repealing Directive 95/46/EC; General Data Protection Regulation (GDPR/AVG) and the Dutch Implementation Act "Uitvoeringswet Algemene verordening gegevensbescherming" (UAVG, as the study did not directly concern humans or animals, and did not offend the privacy rights of any concerned party).

The research team of the University of Groningen, that launched the study is trained to practice scientific integrity and lead this study in line with the university codes of conduct:
- Netherlands Code of Conduct for Research Integrity
- University of Groningen (UG) Academic Integrity Code 2020
- Regulations for the Protection of Academic Integrity UG

Each participant of the study provided informed written consent within the questionnaires which were accompanied with a research statement describing the purpose of the study and how the data would be used.
